# Secreted Frizzled Related Protein 3 in Chronic Heart Failure: Analysis from the Controlled Rosuvastatin Multinational Trial in Heart Failure (CORONA)

**DOI:** 10.1371/journal.pone.0133970

**Published:** 2015-08-19

**Authors:** Erik Tandberg Askevold, Lars Gullestad, Ståle Nymo, John Kjekshus, Arne Yndestad, Roberto Latini, John G. F. Cleland, John J. V. McMurray, Pål Aukrust, Thor Ueland

**Affiliations:** 1 Department of Cardiology, Oslo University Hospital Rikshospitalet, Oslo, Norway; 2 Research Institute for Internal Medicine, Oslo University Hospital Rikshospitalet, Oslo, Norway; 3 Section of Clinical Immunology and Infectious Diseases, Oslo University Hospital Rikshospitalet, Oslo, Norway; 4 Faculty of Medicine, University of Oslo, Oslo, Norway; 5 K. G. Jebsen Cardiac Research Centre and Center for Heart Failure Research, Oslo, Norway; 6 K. G. Jebsen Inflammatory Research Center, Oslo, Norway; 7 K. G. Jebsen Thrombosis Research and Expertise Center, University of Tromsø, Tromsø, Norway; 8 IRCCS-Istituto di Ricerche Farmacologiche “Mario Negri”, Milano, Italy; 9 Department of Cardiology, Hull York Medical School, University of Hull, Castle Hill Hospital, Kingston-upon-Hull, United Kingdom; 10 British Heart Foundation Glasgow Cardiovascular Research Centre, University of Glasgow, Glasgow, United Kingdom; I2MC INSERM UMR U1048, FRANCE

## Abstract

**Background:**

We have previously demonstrated an association between increased sFRP3 expression and adverse outcome in a population of HF irrespective of cause and left ventricular ejection fraction. In this study we evaluated the prognostic value of sFRP3 in older patients with chronic systolic HF of ischemic origin.

**Methods:**

We evaluated sFRP3, by tertiles, as a risk factor for the primary endpoint (cardiovascular [CV] mortality, nonfatal myocardial infarction, nonfatal stroke), all-cause mortality, CV mortality, death from worsening HF (WHF), any coronary event, including sudden death, as well as hospitalizations for CV causes and WHF in 1444 patients from the CORONA population, randomly assigned to 10 mg rosuvastatin or placebo.

**Results:**

Kaplan-Meier curves for the primary endpoint, as well as all-cause- and CV mortality revealed a markedly better survival for patients with sFRP3 levels in the middle tertile of compared to the 1^st^ and 3^rd^ tertile. In multivariable Cox-regression, after full adjustment including high-sensitive CRP and NT-proBNP, a lower event rate for the primary end point, all cause and CV mortality was observed for patients with tertile 2 sFRP3 levels (HR 0.57 [0.44–0.74], 0.55 [0.44–0.74] and 0.52 [0.39–0.69]; *p*<0.001), as well as for the number of coronary events (HR 0.62 [0.47–0.82], *p* = 0.001) and sudden death (HR 0.55 [0.37–0.82], *p* = 0.002). Applying sFRP3 values to the fully adjusted regression model resulted in highly significant continuous net reclassification improvements for the primary endpoint, all cause and CV mortality, coronary events and sudden death (range 0.24–0.31; *p*≤0.002 for all).

**Conclusions:**

Intermediate serum sFRP3 levels are associated with better survival and fewer CV events than low or high sFRP3 levels, independently of conventional risk factors, in older patients with chronic systolic HF of ischemic origin. Our study suggests that balanced Wnt activity might confer protective effects in a clinical HF setting.

**Trial Registration:**

http://www.clinicaltrials.gov NCT00206310

## Introduction

The wingless (Wnt) signaling pathway regulates a multitude of essential cellular processes during embryonic development [1] and well-orchestrated Wnt signaling is necessary for proper heart formation [2]. Wnt activity is normally low and tightly regulated in the adult organism [3], but when dysregulated upon pathological stress or injury, both hypo- and hyper-activity of Wnt signaling has been associated with a wide variety of clinical diseases, including cardiovascular (CV) disorders [4–8]. Secreted modulators regulate both canonical (β-catenin dependent) and non-canonical (β-catenin independent) Wnt signaling at the surface membrane. The secreted frizzled related proteins (sFRPs) bind directly to Wnt ligands in the extracellular space, potentially interfering with both canonical and non-canonical Wnt pathways [9,10]. Several experimental studies have indicated beneficial effects of sFRP1 and sFRP2 on myocardial remodeling [10–15], but few studies have examined the role of sFRP3 in these processes. We recently reported increased left ventricular (LV) mRNA levels of sFRP3 and non-canonical Wnt ligands in end-stage clinical heart failure (HF), with reversible expression patterns upon hemodynamic unloading following LV assist device treatment [16]. In vitro, we identified increased LV wall tension as a potential activator of sFRP3 expression and release. However, a definitive role of sFRP3 in HF development and progression remains unconfirmed.

Secreted Wnt modulators (e.g. sFRP3) are measurable in the systemic circulation and elevated serum and plasma levels have been associated with disease progression and response to therapy in both atherosclerosis and malignant disease [7,17,18]. In a recent report we found an association between serum sFRP3 levels and mortality in a large HF population of mixed etiology, i.e. the GISSI-HF-HF trial [16]. In the present study we investigated the prognostic significance of circulating sFRP3 in patients with chronic systolic HF population of purely ischemic etiology, i.e. a sub-study of patients enrolled in the Controlled Rosuvastatin Multinational Trial in HF (CORONA) [19].

## Methods

### Patients and Study Procedures

Clinical trials identifier: NCT00206310. The trial complied with the Declaration of Helsinki and was approved by the Ethics Committees of the participating hospitals. All patients provided written informed consent. Ethics committee/institutional review board: Regional Etiksprövningskommitten I Göteborg, Sahlgrenska Akademin, Mediniargatan 3, Plan 5. Diary number: Ö284-03.

The name of the ethics committees from any of the participating hospitals (378) can be provided on request. Name of study locations are added at the bottom. The design and principal findings of CORONA have been reported in detail [19]. Briefly, patients ≥60 years of age with chronic HF attributed to ischemic heart disease, defined as (i) medical history or ECG signs of myocardial infarction (MI) or (ii) other data suggesting an ischemic etiology (e.g. wall motion disturbances on echocardiography or history of other occlusive atherosclerotic disease [i.e. earlier stroke, intermittent claudication, percutaneous coronary intervention (PCI)]), who were in New York Heart Association (NYHA) class II-IV, with a LV ejection fraction (LVEF) ≤40% (≤35% if NYHA II), were eligible for inclusion. All patients provided written informed consent. Patients (n = 1444) were randomly assigned to rosuvastatin 10 mg/day (n = 727) or matching placebo (n = 717), once-daily. The present study was an optional, predefined sub-study of the main CORONA trial which included patients from centers capable of collecting the necessary blood samples. Although in general similar to the main CORONA study, there were some modest statistical differences in the baseline characteristics between this sub-study and the complete study population as reported previously [20].

### Study outcomes and definitions

The primary predefined outcome was the composite of death from CV causes, non-fatal MI, and non-fatal stroke, analyzed as time to the first event. The secondary predefined outcomes were a) all-cause mortality, b) CV mortality (including cause-specific CV death), c) any coronary event (defined as sudden death, fatal or non-fatal MI, performance of PCI or coronary artery bypass graft surgery [CABG], ventricular defibrillation by an implantable cardioverter-defibrillator [ICD], resuscitation from cardiac arrest, or hospitalization for unstable angina pectoris), d) the number of hospitalizations for CV causes, and e) hospitalization for worsening HF (WHF). The definition and adjudication of all outcomes have been described in detail previously, as have data on C-reactive protein (CRP) and N-terminal pro-B-type natriuretic peptide (NT-proBNP) [19,21–23].

### Blood sampling and biochemical analyses

sFRP3 was measured from blood samples taken after an overnight fast. All other blood samples were non-fasting and analyzed on fresh samples at a central laboratory (Medical Research Laboratories, Zaventem, Belgium). NT-proBNP was analyzed using commercially available assay (Roche Diagnostics, Basel, Switzerland). An immunonephelometric high-sensitivity method was used to measure CRP (Dade Behring, Atterbury, UK; sensitivity 0.04 mg/L). Serum sFRP3 was measured by enzyme immunoassay (R&D Systems, Minneapolis, MN) as validated previously [16].

### Statistical analysis

For all baseline variables, differences between middle-tertile sFRP3 values and the combination of the highest- and lowest tertile were tested with Student’s t-test for normally distributed variables, Fisher’s exact test for categorical data, and Wilcoxon rank-sum test for non-normally distributed variables. Trends over sFRP3 tertiles were tested with the Cuzick extension of the Wilcoxon rank-sum test, and all baseline variables with a p-value for trend <0.05 were included in a multivariable analysis to identify degree of association with sFRP3. All survival analyses were conducted using the Cox proportional hazard regression model. A restricted cubic spline (RCS) analysis with three knots was undertaken on the outcome all-cause mortality to assess linearity of risk. The RCS analysis revealed a U-shaped curve with lower risk for patients in the middle tertile of sFRP3 concentration corresponding approximately to the pattern seen in Kaplan-Meier plots for all-cause and CV mortality. Therefore, in multivariate analyses, sFRP3 was included as a categorical (by tertiles) variable to a version of the three stage model described elsewhere [23], which included mainly clinical variables at step one (LVEF, NYHA class, age, body mass index [BMI], diabetes mellitus [DM], sex, intermittent claudication, and heart rate [HR]). At step two, estimated glomerular filtration rate (eGFR) and apolipoprotein (Apo) B/ApoA-1 ratio were included in the model, and finally, at stage 3, the log-transformed serum concentrations of NT-proBNP and CRP were included. Harrel’s C-statistic was calculated for all endpoints using the full model with and without sFRP3, and the difference between the C-statistics was estimated. We implemented a jack-knife cross-validation approach to correct for over-optimism associated with validating a model in the same material from which it is developed. In this approach predictions for each observation were obtained from models developed on the remaining observations. These cross-validated probabilities were used to calculate jack-knife C-statistics. Calculation of the net reclassification improvement (NRI) is increasingly being used to evaluate the prognostic usefulness of a biomarker [24]. When no established risk categories exist, the use of a category-free NRI has been advocated [25]. We therefore calculated the category-free NRI after adding sFRP3 to the full model. Confidence intervals and p-values for NRI were determined by boot-strapping with 2000 repetitions. A two-sided p-value <0.05 was considered to be significant, except for interaction terms, for which p-values <0.10 were accepted. All statistical analyses were performed using STATA version 11 for Windows (StataCorp, College Station, TX). The authors had full access to and take responsibility for the integrity of the data. All authors have read and agreed to the manuscript as written.

## Results

Serum concentrations of sFRP3 were measured at baseline in 1444 patients. Patient characteristics according to tertile values of sFRP3 are shown in Table 1. Patients in the top tertile of sFRP3 were more likely to be in atrial fibrillation (AF), take digoxin and had more frequent intermittent claudication. They also had higher NT-proBNP, more use of diuretics and aldosterone antagonists and had slightly worse NYHA functional classification. In addition patients in the top tertile smoked less, had lower total cholesterol levels and lower renal function. Patients in the middle sFRP3 tertile had higher LVEF and significantly lower CRP levels than patients in the top and bottom tertiles. LVEF and CRP were the only variables associated with having sFRP3 in the second tertile in a logistic regression analysis, but with very modest correlation coefficients (r = 0.02, p = 0.034 and r = 0.12, p = 0.012 respectively).

**Table 1:** Clinical and biochemical baseline characteristics stratified by tertile values of sFRP3.

Variable	Total population	sFRP3 tertile 1st	tertile 2nd	tertile 3rd	*P*-value Trend	*P*-value2^nd^
**Age, years**	71.8±6.9	71.8±7.1	71.7±6.7	72.0±6.7	0.587	0.583
**Female sex**	338 (23.4)	118 (24.5)	102 (21.2)	118 (24.5)	0.986	0.167
**NYHA class**					0.015	0.560
**II**	464 (32.1)	174 (36.1)	149 (31.0)	141 (29.3)		
**III**	963 (66.7)	304 (63.1)	328 (68.2)	331 (68.8)		
**IV**	17 (1.2)	4 (0.8)	4 (0.8)	9 (1.9)		
**Ejection Fraction**	0.32±0.07	0.31±0.07	0.32±0.06	0.31±0.07	0.846	0.017
**BMI, kg/m2**	27.2±4.6	27.2±4.8	27.3±4.4	27.3±4.5	0.970	0.764
**SBP, mmHg**	129±16	130±17	130±15	129±16	0.221	0.548
**DBP, mmHg**	77±9	77±9	77±9	77±9	0.792	0.488
**Heart rate, b.p.m**	70.9±10.8	70.5±10.1	71.1±11.1	71.1±11.2	0.561	0.626
**Smoking**	175 (12.1)	82 (17.0)	50 (10.4)	43 (8.9)	0.000	0.171
**Treatment n = (rosuvastatin/placebo)**	727/717	237/245	235/246	245/236	0.785	0.669
**Medical history**						
**Myocardial infarction**	909 (63.0)	307 (63.7)	318 (66.1)	284 (59.0)	0.136	0.083
**Angina pectoris**	1065 (73.8)	363 (75.3)	352 (73.2)	350 (72.8)	0.369	0.751
**Other atherosclerotic disease**	287 (19.9)	90 (18.7)	94 (19.5)	103 (21.4)	0.287	0.834
**PCI or CABG**	300 (20.8)	93 (19.3)	108 (22.5)	99 (20.6)	0.622	0.271
**Hypertension**	1000 (69.3)	323 (67.0)	337 (70.1)	340 (70.7)	0.217	0.672
**Diabetes Mellitus**	378 (26.2)	126 (26.1)	138 (28.7)	114 (23.7)	0.390	0.128
**Atrial Fibrillation/Flutter**	321 (22.2)	81 (16.8)	107 (22.2)	133 (27.7)	<0.001	1.000
**Stroke**	173 (12.0)	65 (13.5)	53 (11.0)	55 (11.4)	0.327	0.441
**Intermittent Claudication**	154 (10.7)	43 (8.9)	49 (10.2)	62 (12.9)	0.046	0.718
**Laboratory measurements**						
**Total cholesterol, mmol/L**	5.23±1.09	5.34±1.13	5.17±0.99	5.17±1.15	0.041	0.170
**LDL, mmol/L**	3.65±0.97	3.71±0.99	3.59±0.99	3.64±0.94	0.286	0.133
**HDL, mmol/L**	1.23±0.34	1.26±0.36	1.22±0.33	1.22±0.34	0.108	0.477
**Triglycerides, mmol/L**	2.01±1.40	2.06±1.51	1.95±1.26	2.02±1.41	0.855	0.239
**Apo B/Apo A-1 value**	0.89±0.25	0.89±0.25	0.87±0.24	0.90±0.26	0.494	0.158
**eGFR**_**MDRD**_ **(mL/min/1.73 m** ^**2**^ **)**	57.4±14.3	58.1±14.6	58.0±13.9	56.2±14.3	0.057	0.270
**NT-pro-BNP, pmol/L**	162 (61–344)	141 (53–322)	164 (64–330)	187 (74–420)	0.005	0.959
**CRP, mg/L**	3.7 (1.6–7.7)	3.6 (1.6–7.9)	3.2 (1.5–6.8)	4.4 (1.9–8.0)	0.152	0.007
**sFRP3, pg/mL**	1190 (853–1760)	729 (30–962)	1191 (963–1524)	2149 (1525–2530)	0.000	0.000
**Medication**						
**Diuretics**					0.006	0.249
**Loop or Thiazide**	1099 (76.1)	369 (76.6)	354 (73.6)	376 (78.2)		
**Both**	157 (10.9)	40 (8.3)	60 (12.5)	57 (11.9)		
**Aldosterone antagonist**	527 (36.5)	161 (33.4)	174 (36.2)	192 (39.9)	0.036	0.862
**ACE inhibitor**	1162 (80.5)	388 (80.5)	387 (80.5)	387 (80.5)	0.987	1.000
**ARB**	147 (10.2)	53 (11.0)	48 (10.0)	46 (9.6)	0.462	0.926
**Beta blocker**	1100 (76.2)	357 (74.1)	369 (76.7)	374 (77.8)	0.179	0.744
**Digitalis glycoside**	416 (28.8)	119 (24.7)	140 (29.1)	157 (32.6)	0.006	0.902

NT-proBNP and CRP are displayed as median value (interquartile range). Other variables are shown as number (percentage of total) or as mean (standard deviation) where appropriate. *P*-value Trend, *p*-value for trend across all tertiles; *P*-value 2^nd^, *p*-value for 2^nd^ tertile compared to 1^st^ and 3^rd^ tertile combined.NYHA, New York Heart Association; BMI, body mass index; SBP, systolic blood pressure; DBP, diastolic blood pressure; PCI, percutaneous coronary intervention; CABG, coronary artery bypass grafting; LDL, low-density lipoprotein; HDL, high-density lipoprotein; ApoB, apolipoprotein B; ApoA-1, apolipoprotein A-1; eGFR, estimated glomerular filtration rate; MDRD, modification of diet in renal disease; CRP, C-reactive protein; NT-proBNP, amino-terminal pro-brain natriuretic peptide; sFRP3, secreted frizzled related protein 3; ACE, angiotensin converting enzyme; ARB, angiotensin II receptor blocker.

### sFRP3 levels and association with outcomes

**Fig 1:**
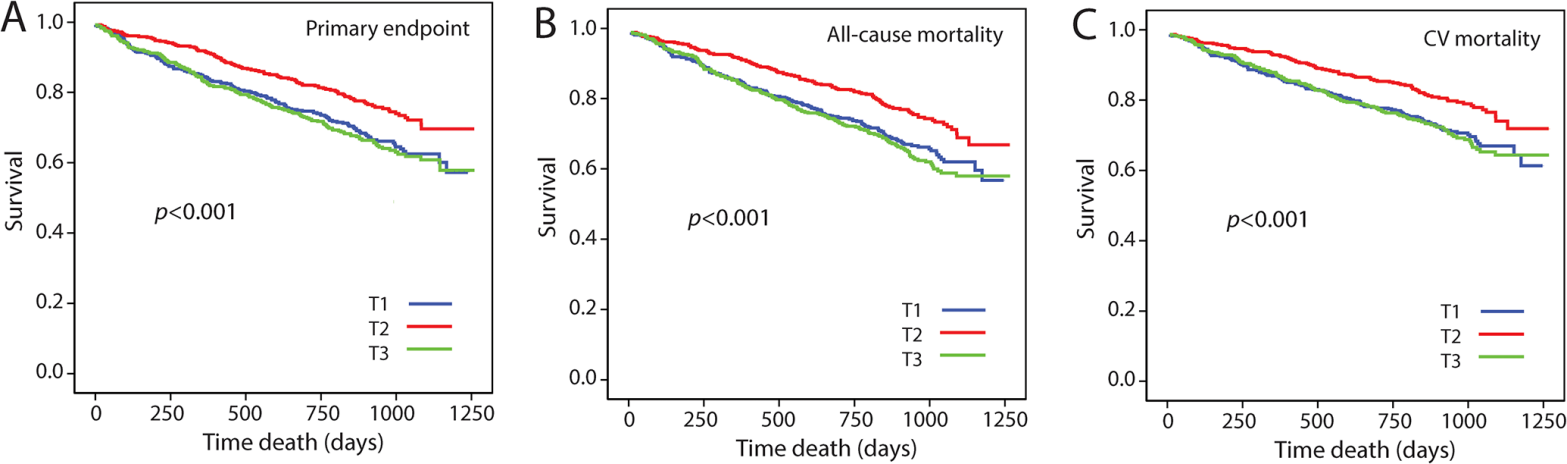
Kaplan-Meier curves for the primary end point (panel A), as well as for all-cause (B) and CV (C) mortality according to tertile sFRP3 concentration. T1, lowest tertile serum sFRP3; T3, highest tertile serum sFRP3. Patients with T2 sFRP3 showed a markedly better outcome than patients in T1 and T2; *p*<0.001 for the primary end point and all-cause mortality, *p*<0.002 for CV mortality.

During a median follow-up of 955 (inter-quartile range 817–1103) days, 421 patients died. Kaplan-Meier plots for the primary end point, as well as for all-cause and CV mortality revealed a markedly poorer outcome for patients in the highest and lowest tertile of sFRP3 concentration compared to the middle tertile (Fig 1). A restricted cubic spline analysis confirmed non-linearity of risk, with a U-shaped curve corresponding approximately to a tertile division of the patient population. Subsequent analyses were therefore undertaken on sFRP3 tertiles. Unadjusted Cox proportional hazard regression models displayed significant associations between baseline sFRP3 levels and the primary endpoint, all-cause and CV mortality, sudden death and coronary events (Table 2). The hazard ratios varied from 0.76 for coronary events to 0.64 for death from CV causes. Baseline sFRP3 was not associated with death from WHF or hospitalizations, irrespective of cause (Table 2).

**Table 2:** Multivariable analysis of intermediate levels of sFRP3 as a predictor of outcome.

sFRP3	Events	HR (95% CI)	*p*-value	Wald	C index, Δ	NRI
**Primary end point**					-0.012 (0.034)	0.26 (<0.001)
**Unadjusted**	406	0.66 (0.53–0.82)	<0.001	13.50		
**Step 1**	406	0.65 (0.52–0.81)	<0.001	14.50		
**Step 2**	404	0.66 (0.53–0.82)	<0.001	13.40		
**Step 3**	315	0.57 (0.44–0.74)	<0.001	18.60		
**All-cause mortality**					-0.0096 (0.055)	0.28 (<0.001)
**Unadjusted**	421	0.66 (0.53–0.82)	<0.001	14.20		
**Step 1**	421	0.65 (0.52–0.81)	<0.001	15.00		
**Step 2**	418	0.65 (0.52–0.82)	<0.001	14.20		
**Step 3**	327	0.55 (0.42–0.71)	<0.001	21.40		
**CV mortality**					-0.012 (0.045)	0.31 (<0.001)
**Unadjusted**	342	0.64 (0.51–0.82)	<0.001	12.70		
**Step 1**	342	0.64 (0.51–0.82)	<0.001	12.70		
**Step 2**	340	0.65 (0.51–0.83)	0.001	11.60		
**Step 3**	264	0.52 (0.39–0.69)	<0.001	19.90		
**Death from WHF**					-0.0045 (0.44)	0.17 (0.42)
**Unadjusted**	104	0.72 (0.47–1.10)	0.127	2.32		
**Step 1**	104	0.72 (0.47–1.11)	0.140	2.18		
**Step 2**	104	0.73 (0.47–1.12)	0.149	2.08		
**Step 3**	80	0.59 (0.36–0.99)	0.045	4.02		
**Sudden death**					-0.0066 (0.40)	0.25 (0.002)
**Unadjusted**	191	0.69 (0.51–0.96)	0.025	5.01		
**Step 1**	191	0.69 (0.50–0.95)	0.022	5.21		
**Step 2**	189	0.70 (0.51–0.97)	0.030	4.71		
**Step 3**	149	0.55 (0.37–0.80)	0.002	9.89		
**Coronary end point**					-0.0060 (0.37)	0.24 (<0.001)
**Unadjusted**	326	0.76 (0.55–0.90)	0.005	7.88		
**Step 1**	326	0.71 (0.55–0.90)	0.005	7.84		
**Step 2**	322	0.71 (0.55–0.91)	0.006	7.52		
**Step 3**	254	0.62 (0.47–0.82)	0.001	11.4		
**Hospitalization, any cause**					0.00082 (0.10)	0.018 (0.73)
**Unadjusted**	814	0.91 (0.79–1.05)	0.205	1.60		
**Step 1**	812	0.91 (0.79–1.06)	0.225	1.78		
**Step 2**	805	0.91 (0.78–1.05)	0.183	1.78		
**Step 3**	655	0.96 (0.81–1.13)	0.623	0.24		
**Hospitalization, CV cause**					0.00083 (<0.001)	0.028 (0.58)
**Unadjusted**	613	0.95 (0.80–1.12)	0.523	0.41		
**Step 1**	611	0.95 (0.81–1.13)	0.595	0.28		
**Step 2**	606	0.95 (0.80–1.13)	0.565	0.33		
**Step 3**	499	0.99 (0.82–1.19)	0.877	0.02		
**Hospitalization from WHF**					0.00074 (0.33)	0.042 (0.47)
**Unadjusted**	334	0.97 (0.78–1.22)	0.816	0.054		
**Step 1**	333	0.97 (0.77–1.21)	0.769	0.086		
**Step 2**	331	0.95 (0.75–1.19)	0.639	0.22		
**Step 3**	275	0.93 (0.73–1.20)	0.601	0.27		
**Death or hospitalization from WHF**					0.00081 (0.53)	0.089 (0.21)
**Unadjusted**	371	0.93 (0.75–1.16)	0.528	0.40		
**Step 1**	370	0.93 (0.75–1.15)	0.498	0.46		
**Step 2**	368	0.91 (0.73–1.13)	0.401	0.71		
**Step 3**	300	0.90 (0.71–1.15)	0.410	0.68		

sFRP3, 2^nd^ tertile *vs*. 1^st^ and 3^rd^ tertile, as predictor of outcome. All Hazard Ratios (HR) are given as HR (95% confidence interval). C index, Δ; difference in C index between fully adjusted model with and without inclusion of sFRP3, corresponding (*p*-value). Net Reclassification Improvement (NRI); calculated from C-indexes for fully adjusted models with and without inclusion of sFRP3, corresponding (*p*-value). Unadjusted (n = 1444). The models are adjusted as follows: Step 1 (n = 1441): Ejection fraction, New York Heart Association functional class, age, body mass index, diabetes mellitus, sex, intermittent claudication and heart rate. Step 2 (n = 1428): All variables from Step 1 as well as ApoB/Apo A-1 ratio and estimated glomerular filtration rate. Step 3 (1194): all variables from Step 2 as well as C-reactive protein and amino-terminal pro B-type natriuretic peptide. CV, cardiovascular; WHF, worsening heart failure.

When adjusting for demographic variables (step 1) in a three step multivariable analysis, sFRP3 was still associated with the primary endpoint, all-cause mortality, death due to CV cause, sudden death and coronary events (Table 2). These associations remained significant after adjusting for ApoB/ApoA-1 ratio and eGFR (step 2). When correcting for NT-proBNP and CRP (step 3), sFRP3 remained a strong predictor for the primary endpoint, overall mortality, death from CV cause, sudden death and coronary events, with little change in hazard ratios from the unadjusted model (Table 2). As seen in Table 3, addition of sFRP3 in the multivariable analyses did not alter the effects of other variables, suggesting incremental value of sFRP3 and a non-competing relation to conventional risk factors. This finding is further underscored by highly significant continuous net reclassification improvements (NRI) for the primary endpoint (NRI 0.26, p<0.0001), all-cause mortality (NRI 0.28, p<0.000001), death from CV cause (NRI 0.31, p<0.000001), as well as for coronary events (NRI 0.24, p<0.0001) and sudden death (NRI 0.25, p = 0.002) when sFRP3 was added to the fully adjusted models (Table 2). However, the isolated discriminatory properties of sFRP3 were limited (Table 4), indicating that sFRP3 measurements, at present, are mostly of interest in multi-marker risk models and from a mechanistic point of view.

**Table 3:** Effect of sFRP3 on the association between other predictors and outcome.

		All-cause Mortality		CV Mortality	
Variable		Without sFRP3	With sFRP3	Without sFRP3	With sFRP3
**Ejection fraction (x 100)**	HR	1.00 (0.98–1.02)	1.00 (0.98–1.02)	1.00 (0.98–1.02)	1.00 (0.98–1.02)
**Age (x 10)**	HR	1.21 (0.94–1.34)	1.14 (0.95–1.36)	1.09 (0.89–1.33)	1.11 (0.91–1.35)
**Heart rate (x 1/10)**	HR	1.06 (0.96–1.17)	1.07 (0.96–1.18)	1.05 (0.93–1.17)	1.05 (0.94–1.18)
**Female sex**	HR	0.72 (0.54–0.97)	0.69 (0.52–0.92)	0.73 (0.57–1.07)	0.75 (0.55–1.03)
**Diabetes Mellitus**	HR	1.60 (1.25–2.04)	1.61 (1.26–2.05)	1.54 (1.18–2.02)	1.55 (1.18–2.03)
**Intermittent Claudication**	HR	1.46 (1.08–1.99)	1.49 (1.09–2.02)	1.31 (0.92–1.87)	1.34 (0.94–1.92)
**BMI**	HR	0.95 (0.93–0.98)	0.95 (0.93–0.98)	0.96 (0.93–0.99)	0.96 (0.93–0.99)
**NYHA III**	HR	1.15 (0.88–1.49)	1.11 (0.85–1.44)	1.12 (0.83–1.49)	1.07 (0.80–1.44)
**NYHA IV**	HR	1.53 (0.75–3.14)	1.37 (0.66–2.84)	1.52 (0.71–3.28)	1.34 (0.61–2.92)
**eGFR**	HR	0.99 (0.98–1.00)	0.99 (0.98–1.00)	0.99 (0.98–1.00)	0.99 (0.98–1.00)
**Apo B/Apo A-1 ratio**	HR	1.06 (0.67–1.68)	1.04 (0.65–1.64)	1.22 (0.74–2.02)	1.20 (0.72–1.98)
**NT-proBNP**	HR	1.62 (1.45–1.82)	1.63 (1.46–1.83)	1.76 (1.55–2.00)	1.78 (1.56–2.01)
**CRP**	HR	1.19 (1.09–1.31)	1.18 (1.07–1.30)	1.16 (1.04–1.28)	1.14 (1.03–1.27)
**T2 sFRP3**	HR		0.55 (0.42–0.71)		0.52 (0.39–0.69)

BMI, body mass index; NYHA, New York Heart Association; eGFR, estimated glomerular filtration rate; ApoB, apolipoprotein B; ApoA-1, apolipoprotein A-1; NT-proBNP, amino-terminal pro-brain natriuretic peptide; CRP, C-reactive protein; T2 sFRP3, middle tertile secreted frizzled related protein 3.

**Table 4:** Discriminatory properties of sFRP3. Area under curve (AUC) and 95% Confidence interval (CI) of sFRP3 as a categorical (1. and 3. tertile *vs*. 2. tertile) variable, corresponding *p*-value.

End point	AUC	CI	*p*-value
**Primary endpoint**	0.55	0.53–0.57	<0.001
**All-cause mortality**	0.55	0.53–0.57	<0.001
**CV mortality**	0.55	0.55–0.57	<0.001
**Death from WHF**	0.53	0.49–0.58	0.128
**Sudden death**	0.54	0.51–0.57	0.012
**Coronary endpoint**	0.54	0.51–0.56	0.003
**Hospitalization, any cause**	0.51	0.49–0.53	0.238
**Hospitalization, CV cause**	0.51	0.49–0.53	0.545
**Hospitalization due to WHF**	0.50	0.48–0.53	0.910

### Comparison with the GISSI-HF-HF trial

**Fig 2:**
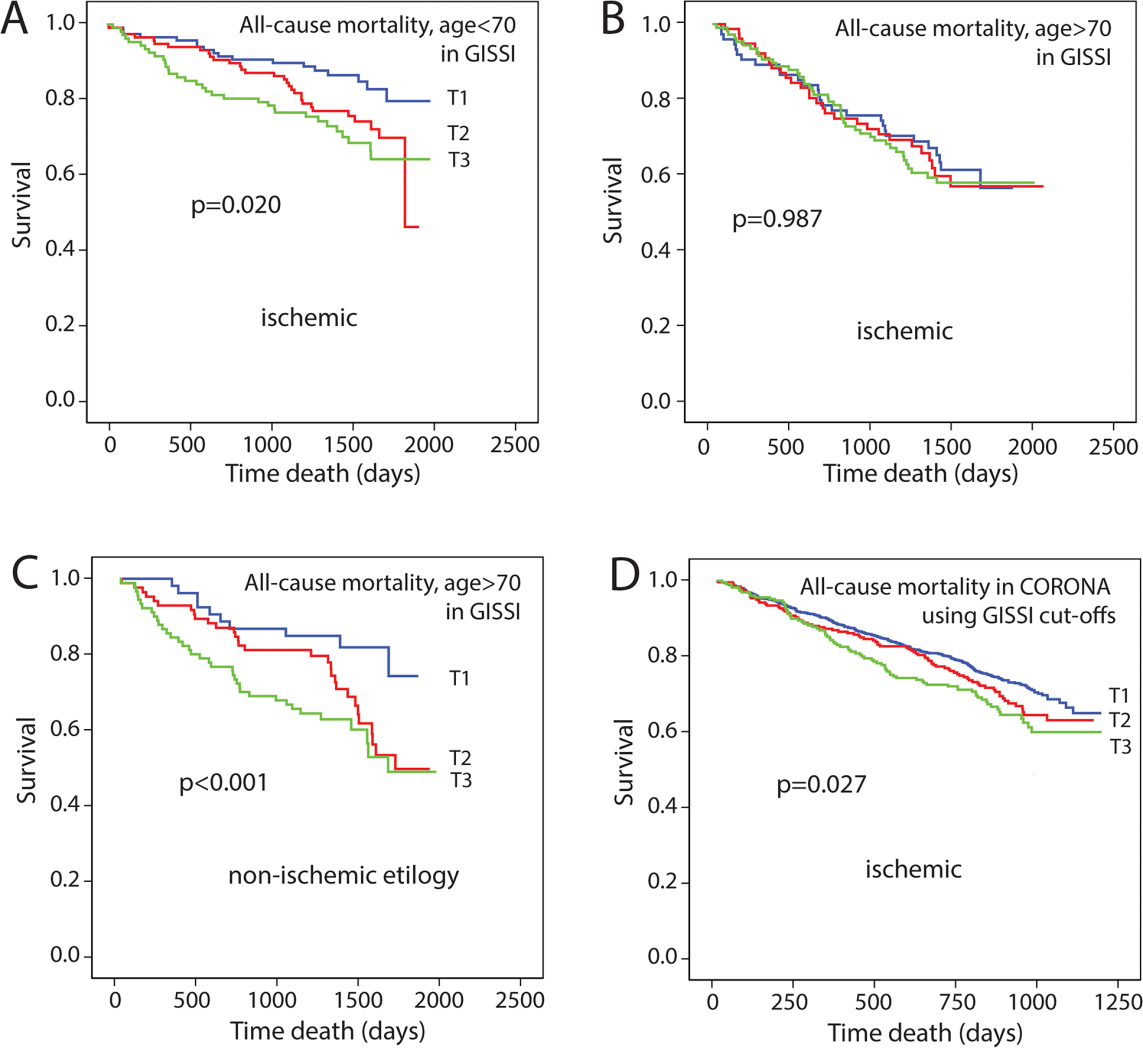
Kaplan Meier plots showing the association between tertiles of sFRP3 and all-cause mortality in the GISSI-HF-HF trial stratified according to age and presence of ischemic heart disease. **A.** ischemic HF <70 years of age **B.** ischemic HF >70 years of age **C.** non-ischemic HF > 70 years **D.** all-cause mortality in CORONA using cut-off derived from the GISSI-HF-HF trial.

These finding are in contrast to a recent study from our group demonstrating a linear increase in risk for outcome in 1202 HF patients with mixed etiology from the GISSI-HF-HF trial [16]. When evaluating the association between sFRP3 and outcome in patients with ischemic etiology in the GISSI-HF trial, a similar association was observed in patients <70 years of age with ischemic etiology (n = 345, Fig 2A), as when all patients in GISSI-HF with both etiologies were included. However, when looking at patients >70 years of age with ischemic etiology (n = 261), no association was observed (Fig 2B), in contrast to non-ischemic patients aged >70 years (n = 215, Fig 2C). In addition, when applying the tertile limits derived from the GISSI-HF population on the CORONA population we found a similar stepwise association with outcome (i.e. all-cause and CV mortality) as observed in GISSI-HF, although weaker (Fig 2D).

### Effect of rosuvastatin treatment on sFRP3 levels and clinical outcomes

Serum sFRP3 concentrations were similar at baseline and three month follow up (continuous variable) both in patients assigned to placebo (n = 717) or rosuvastatin (n = 727). An interaction of sFRP3 and treatment group was not observed for any endpoints.

## Discussion

In this post-hoc analysis from CORONA we found serum concentrations of sFRP3 to be associated with fatal outcomes in a large population of elderly patients with chronic systolic HF of ischemic origin, with a significantly worse prognosis for patients in the first and third tertiles as opposed to those in the second tertile. Hazard ratios attributed to mid-tertile sFRP3 values remained significant and stable for the primary endpoint, all-cause- and CV mortality, sudden death and coronary events also after adjusting for established risk factors, including NT-proBNP and CRP, in a step-wise fashion. These data suggest a biphasic association between sFRP3 and outcome in the CORONA population with high and low levels associated with a poorer prognosis.

We have previously shown elevated sFRP3 levels in the GISSI-HF-HF population of both ischemic and non-ischemic etiologies [16], with unfavorable prognosis associated with increasing sFRP3 concentrations. Recently, Motiwala et al. assessed the predictive value of sFRP3 in 142 patients with HF [26] and found no significant association with mortality, although a trend towards higher levels in patients with a CV event was observed (p = 0.10). Furthermore, no survival analysis was performed and the HF populations differed markedly in size, demographics, endpoint definition and follow-up period making it difficult to compare the studies. Compared to our finding in the GISSI-HF-HF trial [16], the current study partly contradicts these findings by demonstrating a non-linear association between sFRP3 and outcomes in the CORONA population. This discrepancy may partly be explained by different characteristics of the study populations. The CORONA population had substantially lower functional capacity, with ~70% of the patients being in NYHA III-IV compared to 26% in GISSI-HF-HF. Moreover, the CORONA study included only patients with reduced LVEF while patients with both reduced and preserved LVEF were included in GISSI-HF-HF, and kidney function was also lower in the CORONA cohort (eGFR 57 *vs*. 69 mL/min/1.73m^2^). The CORONA population consisted of older patients (71.8±6.9 *vs*. 66.3±10.8 years in GISSI-HF-HF) with HF of ischemic etiology, whereas the GISSI-HF-HF patients had HF of both ischemic and non-ischemic etiology. Indeed, when evaluating mortality in the GISSI-HF-HF trial stratified by etiology and age we found that in contrast to patients with ischemic HF <70 years who demonstrated a more linear association between sFRP3 and outcome, this association was not present in older patients (i.e. >70 years) with ischemic HF. Furthermore, although caution is needed when comparing circulating sFRP3 from different populations analyzed on separate occasions, sFRP3 levels were approximately 50% higher in GISSI-HF-HF compared to CORONA. Thus, the first tertile of GISSI-HF and the second tertile in CORONA had comparable levels and were associated with the best outcome, while tertile 3 in CORONA and tertile 2 and 3 in GISSI-HF represent a further increase in adverse events. Indeed, applying the tertile limits derived from the GISSI-HF population [16] on the CORONA population demonstrated a similar, although weaker, stepwise association with outcome as observed in GISSI-HF.

**Fig 3:**
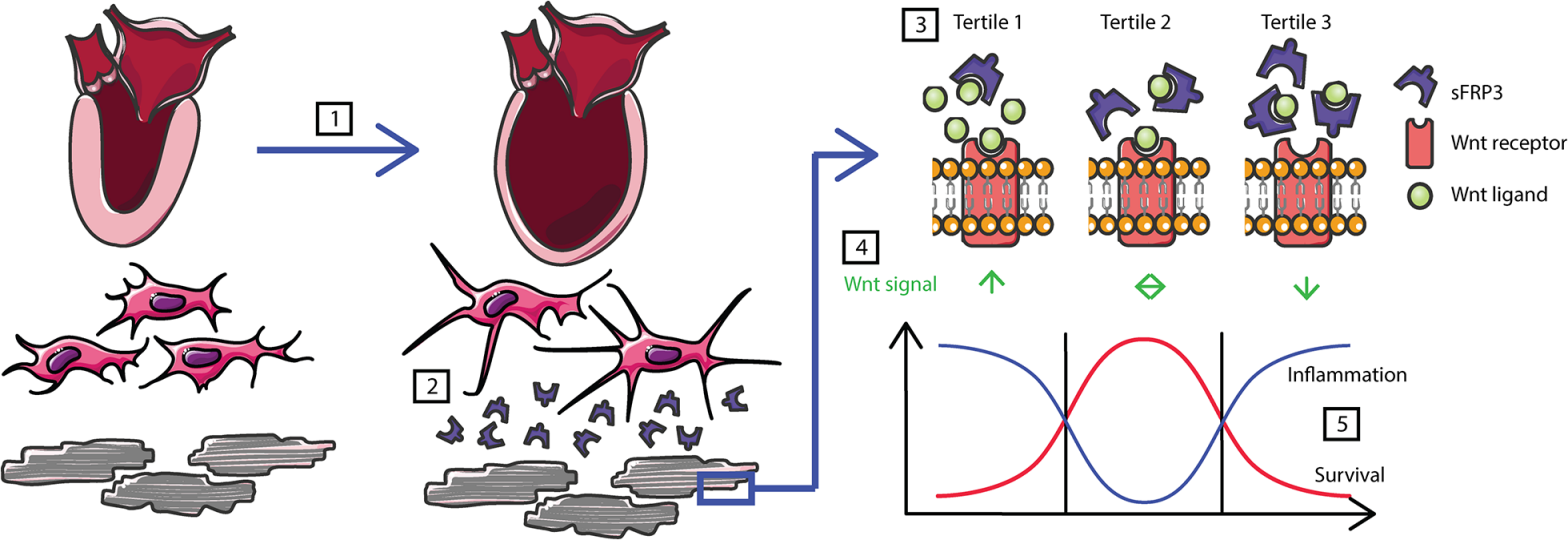
Possible mechanism linking sFRP3 release during LV wall stress and non-linear association with survival. Increased wall stress [1] may induce the release of sFRP3 from fibroblasts [2]. Depending on concentration of sFRP3 [3], this may lead to insufficient, balanced or excess inhibition of the Wnt [4] in the presence of inflammation and lead to a non-linear association with survival [5].

The non-linear associations between sFRP3 levels and outcomes in this study might also be explained by inherent properties of Wnt signaling and the sFRP family of Wnt-modulators. A recent report from our group has indicated that secretion of sFRP3 might be a compensatory mechanism whereby the myocardium seeks to limit increased LV wall stress [16]. Thus, low levels of sFRP3 could reflect inadequate response to pathological Wnt activity following ischemic injury and increased inflammation and high levels of sFRP3 might represent an overshoot of repair mechanisms, which in itself might be deleterious. Patients with intermediate sFRP3 levels were characterized by lower CRP and higher LVEF suggesting that these patients were healthier. Fig 3 illustrates the possible mechanism linking release of sFRP3 during LV wall stress and a non-linear association with outcome. Also, others have reported biphasic effects of sFRPs, potentially reflecting their complex mechanisms of action [10,27,28]. For instance, extracellular sequestering of Wnt ligands and formation of inactive complexes with membrane bound Frizzled receptors (Fzd-R) antagonizes Wnt signaling, whilst simultaneous binding to ligand and receptor and sFRP-sFRP binding, titrating out each other’s activity, favor Wnt signaling [10,27]. Also, Xavier et al. demonstrated that sFRP1either inhibits or enhances canonical Wnt signaling depending on concentration and cellular context [28].

In the current study sFRP3 remained strongly associated with outcomes even after extensive multivariable adjustment, including NT-proBNP, with stable HRs and relatively high Wald scores. Adding sFRP3 to fully adjusted multivariable models produced highly significant NRIs for all fatal outcomes, except for death from WHF, as well as for the coronary endpoint. Although analyses of sFRP3 in the current cohort suggests a significant association with adverse prognosis, the use of the mid-tertile as a biomarker is, at least at present, not suitable for clinical use. Nonetheless, our findings indicate involvement of soluble Wnt modulators in the progression of clinical HF, with a potential complex interaction within the different members of the Wnt family. It is conceivable that a greater understanding of Wnt signaling in HF might provide us with new tools in the therapeutic armamentarium as both over-expression and exogenous administration of sFRPs reduces morbidity and mortality in murine MI models [12,14].

The present study examined multiple end points in a large HF population with a considerable number of events. However, for some subgroup analyses, including analyses of the endpoint Death from WHF and the interaction of sFRP3 with rosuvastatin, there were relatively few events and, therefore, these data should be interpreted cautiously. Our study was performed in trial patients of ≥60 years of age, with LV systolic dysfunction and ischemic heart disease. Thus, the results may not apply to all patients with HF. There are a number of other sFRP’s and other Wnt antagonists that future studies should evaluate as predictors of adverse outcome in HF patients. The relatively poor association with established CV determinants, and current medications in our study, may imply that Wnt signaling is relatively unaffected by state of the art treatment regimens. Thus, the Wnt signaling pathway might represent an untapped therapeutic potential for HF modulation.

In conclusion, mid-tertile serum concentrations of sFRP3 were associated with reduced fatality in a large population of elderly patients with chronic systolic HF of ischemic origin. Hazard ratios attributed to mid-tertile sFRP3 values remained significant and stable also when accounting for established risk factors, including NT-proBNP and CRP. Although the use of sFRP3 as a biomarker in clinical practice is premature, our findings support the involvement of Wnt signaling in HF progression and suggest that this novel pathway might represent an as yet unmodified mechanism in HF development.
